# Cellular and Tissue Mechanisms of Laser-Assisted Regeneration of Tympanic Membrane Perforation

**DOI:** 10.3390/ijms27198906

**Published:** 2026-10-07

**Authors:** Ekaterina Valerievna Blinova, Igor Vyacheslavovich Lunyov, Andrey Igorevich Osipyants, Kirill Dmitrievich Blinov, Anna Vladimirovna Zolotova, Liudmila Mikhailovna Ignatova, Valeriy Michailovich Svistushkin, Valeria Vadimovna Gainutdinova, Mikhail Valerievich Svistushkin, Ekaterina Mikhailovna Kasianenko, Alexander Ivanovich Omelchenko, Aleksey Vasilevich Yuzhakov, Olga Igorevna Baum

**Affiliations:** 1 Department of Clinical Pharmacology and Propaedeutics of Internal Diseases, Sechenov First Moscow State Medical University, Moscow 119991, Russia; blinov_k_d@staff.sechenov.ru; 2 Engineering Physics Institute for Biomedicine, Moscow Engineering Physics Institute ( National Research Nuclear University), Moscow 115409, Russia; ivlunev@mephi.ru (I.V.L.); osssip@gmail.com (A.I.O.); 3 Dmitry Rogachev National Medical Research Center of Pediatric Hematology, Oncology and Immunology, Laboratory of Pharmacology and Health Technology Assessment, Moscow 117198, Russia; 4 Department of Ear, Nose and Throat Diseases, Sechenov First Moscow State Medical University, Moscow 119991, Russia; zolotova_a_v@staff.sechenov.ru (A.V.Z.); svistushkin_v_m@staff.sechenov.ru (V.M.S.); svistushkin_m_v@staff.sechenov.ru (M.V.S.); 5 National Research Centre “Kurchatov Institute”, Moscow 123182, Russiayuzhalvas@gmail.com (A.V.Y.); baumolga1@mail.ru (O.I.B.)

**Keywords:** tympanic membrane, laser irradiation, photobiomodulation, fibroblasts, tissue regeneration, scratch assay, collagen synthesis, tympanic membrane perforation, infrared laser

## Abstract

Chronic tympanic membrane (TM) perforations remain a significant clinical problem due to limited spontaneous healing and the drawbacks of surgical interventions. The present study aimed to evaluate the effects of low-intensity infrared laser irradiation on cellular mechanisms of regeneration and to assess its therapeutic potential in TM repair. Primary fibroblast cultures isolated from chinchilla tissue were exposed to laser radiation at wavelengths 1560 nm and 1450 nm. Cellular responses were analyzed using MTT assay, scratch test, Ki-67 immunostaining, and ELISA for collagen type II. In addition, an in vivo model of chronic TM perforation in chinchillas was used to validate the regenerative effects under physiological conditions. Laser irradiation was associated with accelerated scratch closure and increased extracellular matrix production under selected irradiation conditions, whereas metabolic and proliferative indices decreased over time, most likely reflecting culture confluence and maturation rather than cytotoxicity. In vivo, complete closure of tympanic membrane perforations was observed in all laser-treated ears in the context of collagen sponge-assisted repair, whereas scaffold-only control perforations remained unclosed. These findings support the potential of low-intensity infrared laser irradiation as a minimally invasive adjunct to scaffold-assisted tympanic membrane repair. However, the proposed involvement of endogenous progenitor cell populations should be regarded as a hypothesis requiring further histological and marker-based validation.

## 1. Introduction

One of the urgent tasks of modern otorhinolaryngology and biomedicine is the development of effective methods for stimulating the regeneration of the tissues of the middle ear, in particular the tympanic membrane (TM). While acute perforations typically undergo spontaneous closure within 7–10 days, chronic defects persist for at least three months, creating conditions increasing the probability of middle ear infection and permanent hearing loss. This results in substantial deterioration of quality of life, social maladaptation, and communication limitations for affected patients [1,2].

Although tympanoplasty and myringoplasty are standard procedures for TM perforations, they carry notable disadvantages. These include the potential for graft failure and subsequent reperforation. Additionally, these approaches often necessitate prolonged hospitalization and extended recovery periods, resulting in significant temporary disability and healthcare costs [3,4,5,6].

This has led to a growing interest in alternative closure methods. These tissue-engineered approaches often combine various scaffolds, growth factors, and stem cells to promote the regeneration of the tympanic membrane [7].

In this regard, physical methods of stimulating reparative processes are of particular interest, among which increasing attention is being paid to low-intensity pulsed-periodic IR-laser exposure for modifying the structure and reconstructing biological tissues, associated with the absence of destruction and coagulation of the biological object and maintaining its functionality [8]. As an example of such technologies, it is necessary to mention the technology of laser treatment of laryngeal stenosis by means of costal cartilage transplantation developed and brought to clinical practice by our research team [8,9], as well as the technology of laser exposure to articular cartilage for the purpose of regenerating cartilaginous tissue [10,11].

In recent years, a significant amount of data has been accumulated on the biostimulating effect of laser radiation on connective tissue cells, including fibroblasts, chondrocytes, keratinocytes, and endothelial cells [12,13,14,15,16,17]. The mechanisms of this effect are associated with the activation of mitochondrial enzymes, changes in the potential of the cell membrane, increased ATP synthesis, and regulation of the expression of genes responsible for the proliferation and synthesis of the extracellular matrix (ECM) [15,16,17,18].

One of the best-characterized molecular mechanisms of photobiomodulation involves the absorption of light energy by cytochrome c oxidase in mitochondria, resulting in increased mitochondrial membrane potential and ATP production [19]. The associated changes in redox status, including moderate ROS generation, together with modulation of intracellular Ca^2+^ homeostasis, may initiate early signaling events involved in tissue repair [20]. These primary cellular responses can subsequently activate several intracellular signaling pathways that regulate fibroblast proliferation, migration, and stress responses. In particular, the mitogen-activated protein kinase (MAPK) family, including ERK1/2, JNK, and p38 MAPK, is responsive to PBM in a wavelength- and dose-dependent manner, with changes in protein phosphorylation contributing to the regulation of cell proliferation and migration of fibroblasts [21].

Collagen and elastic fibers, being the primary components of the ECM, dictate biomechanical properties, and can upregulate regenerative processes; these structures are found to be susceptible to thermal effects, induced by laser radiation [22].

Macrophages are recognized for their role in tissue remodeling: the degree and phenotype of these cells determine whether the process will be inflammatory (M1 phenotype) or reparative (M2 phenotype) [23]. Wei-Ting et al., 2021, suggested that near-infrared (NIR) radiation enhances citrate synthase gene expression, inducing macrophage M2 polarization, making them very laser-sensitive targets [24].

Previous studies have identified latent epithelial stem or progenitor cells within the tympanic membrane [25,26,27]. We hypothesize that laser irradiation may act as a stimulating factor for regenerative processes within the tympanic membrane. In particular, laser stimulation may influence endogenous cellular populations involved in TM repair; however, direct verification of this mechanism requires additional marker-based and histological studies [28].

The novelty of the present study lies in the combined evaluation of wavelength- and geometry-dependent infrared laser stimulation in primary chinchilla fibroblasts and in a chronic chinchilla TM perforation model. In contrast to previous photobiomodulation studies, we focused on 1450/1560 nm infrared irradiation, compared single-point and multi-point exposure strategies, and linked in vitro cellular responses with scaffold-assisted in vivo TM repair. The proposed involvement of endogenous TM progenitor zones is considered a working hypothesis rather than a directly demonstrated mechanism.

**Figure 1:**
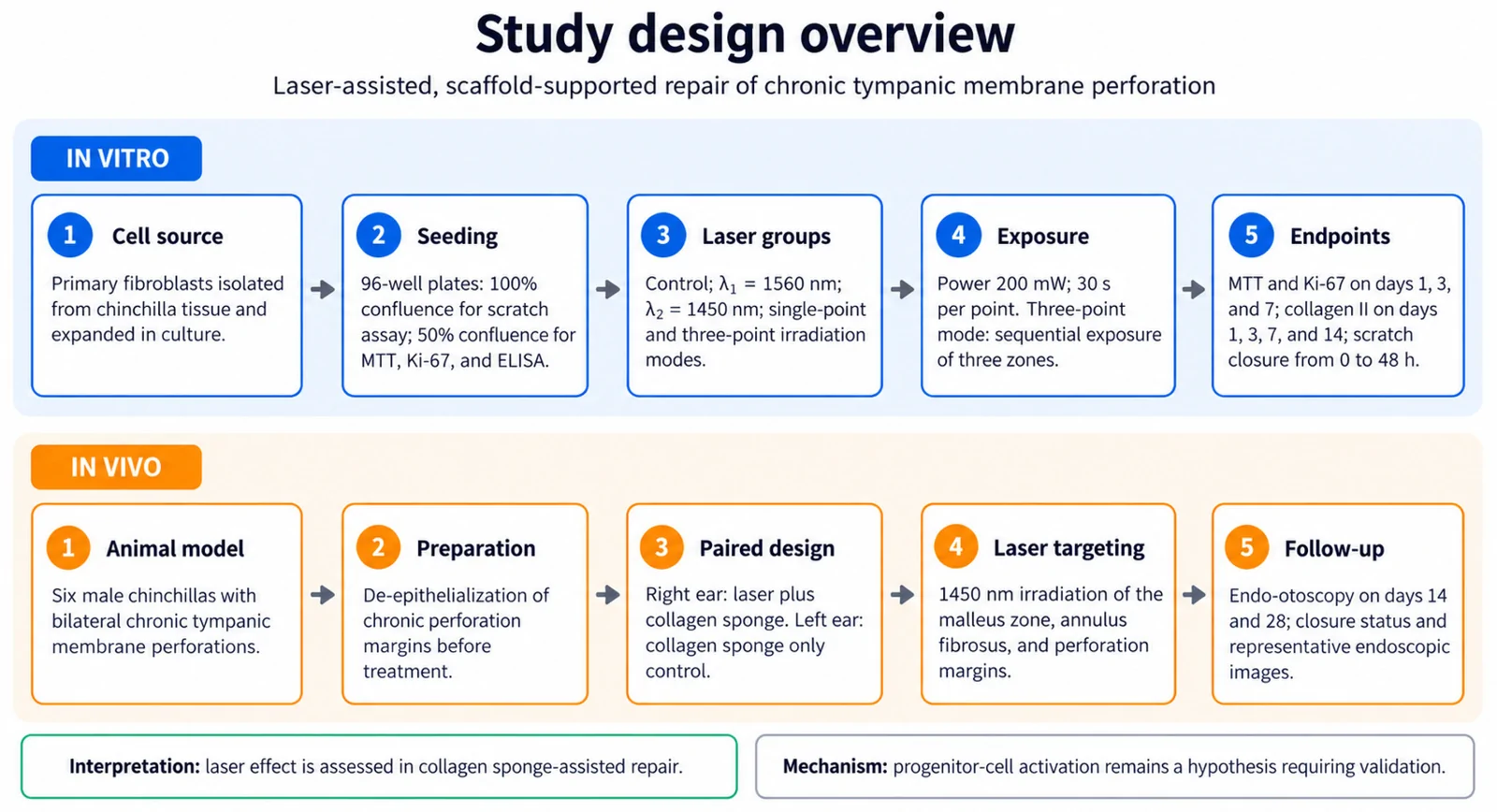
Schematic overview of the experimental design. In vitro, primary chinchilla fibroblasts were irradiated with 1560 nm or 1450 nm infrared laser light using single-point or three-point exposure modes, followed by assessment of metabolic activity, proliferative activity, extracellular matrix production, and scratch closure dynamics. In vivo, bilateral chronic tympanic membrane perforations were created in chinchillas; after de-epithelialization, both ears received a collagen sponge scaffold, while the right ear additionally underwent laser treatment. Endoscopic assessment was performed on postoperative days 14 and 28.

The overall experimental design, including the in vitro and in vivo workflows, treatment groups, irradiation conditions, timing of analysis, and endpoints, is summarized in Figure 1.

## 2. Results

### 2.1. Estimation of the Metabolic Activity: MTT Test

The MTT test (3-(4,5-dimethylthiazol-2-yl)-2,5-diphenyltetrazolium bromide) is one of the most common methods for assessing the metabolic activity and viability of cell cultures. The principle of the method is based on the ability of mitochondrial dehydrogenases of living cells to reduce the yellow tetrazolium dye MTT to a violet crystalline formazane insoluble in water. This process occurs only in metabolically active cells, as it requires a functioning respiratory chain and sufficient levels of intracellular NADH and NADP.

**Figure 2:**
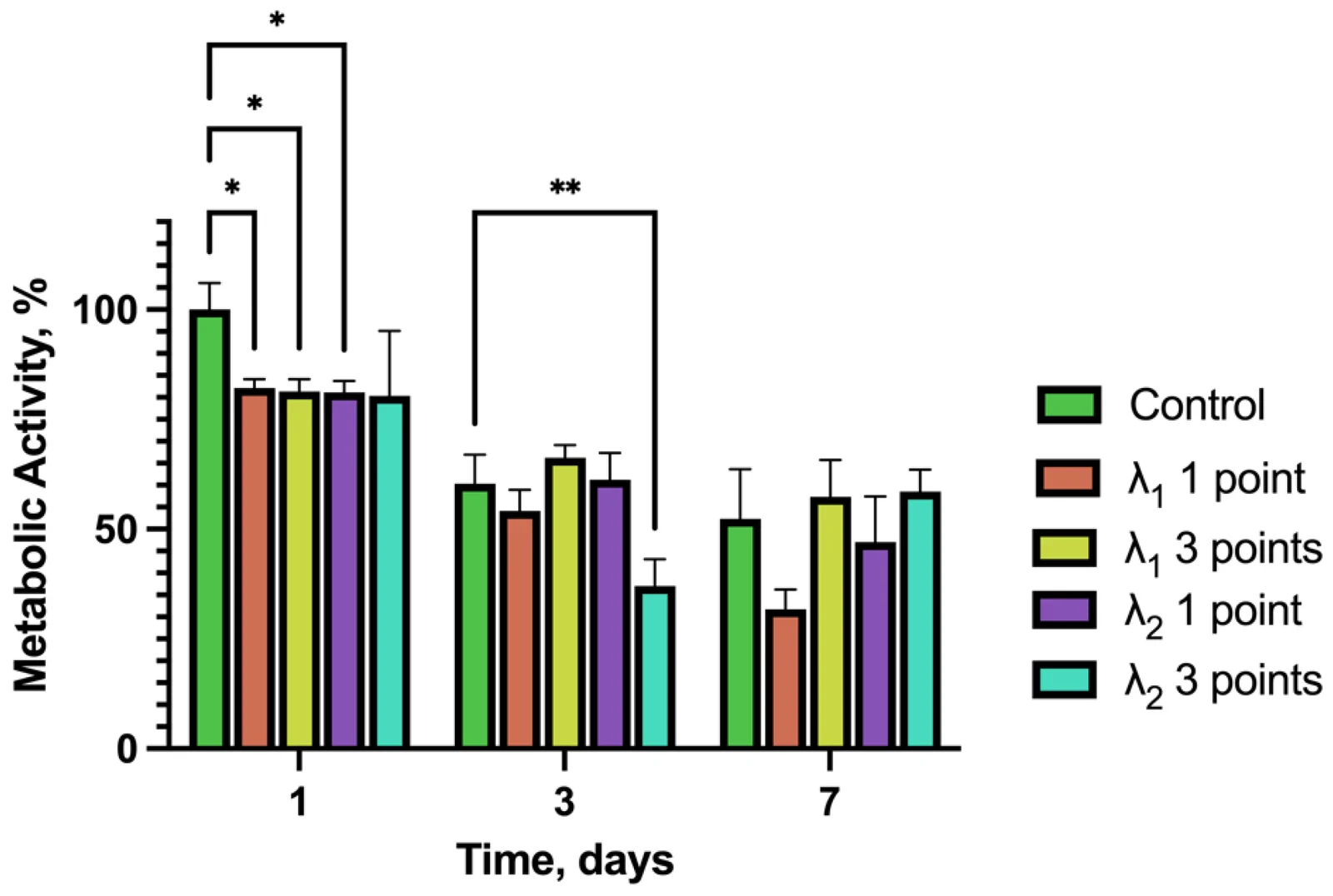
Results of the MTT test on fibroblasts. Data shown are the means ± SD for 3 independent experiments. Statistical significance is indicated as follows: * *p* < 0.05, ** *p* < 0.01 vs. control (ANOVA and Tukey’s HSD tests).

The results of the MTT test, which assessed the metabolic activity of fibroblasts, are shown in Figure 2.

According to the results obtained, there was a gradual decrease in the metabolic activity of fibroblasts, measured using the MTT test, relative to the control group on the first day of the experiment. At an early stage (day 1), the recovery rate of the tetrazolium compound was the highest, indicating high metabolic activity in the cells. By the third day, a moderate decrease in optical density was observed, corresponding to a decrease in the amount of formazan, a product of MTT reduction by mitochondrial enzymes. By the seventh day, the decrease in metabolic activity became more pronounced, however, the morphology of the cell layer remained stable, and the number of living cells did not decrease significantly compared to previous time points.

Such dynamics may be due to the cells reaching their maximum density, a state of complete confluence in which fibroblast growth and division slow down due to contact inhibition. In conditions of high cell layer density, metabolic processes, including mitochondrial respiration, decrease, which is reflected in a decrease in the intensity of MTT recovery. The recorded decrease in optical density at the late stages of the experiment does not indicate a decrease in cell viability, but rather reflects the physiological stabilization of metabolic activity characteristic of a mature monolayer culture.

Statistical processing of the results of the MTT test for fibroblasts showed that on the first day, a decrease in metabolic activity was recorded in many experimental groups compared with the control, and for some samples these differences reached statistical significance (*p* < 0.05). On the third day, the most pronounced decrease in metabolic activity was observed in cells irradiated with the second wavelength in the three-point mode, where the change in metabolic rate was highly reliable (*p* < 0.01), while the remaining groups showed values comparable to the control and did not exceed the limits of statistical error. By the seventh day, all experimental conditions were aligned in terms of optical density, and differences between the irradiated samples and the control were no longer recorded.

### 2.2. Dynamics of Scratch Closure in Fibroblast Monolayers: Scratch Assay

**Figure 3:**
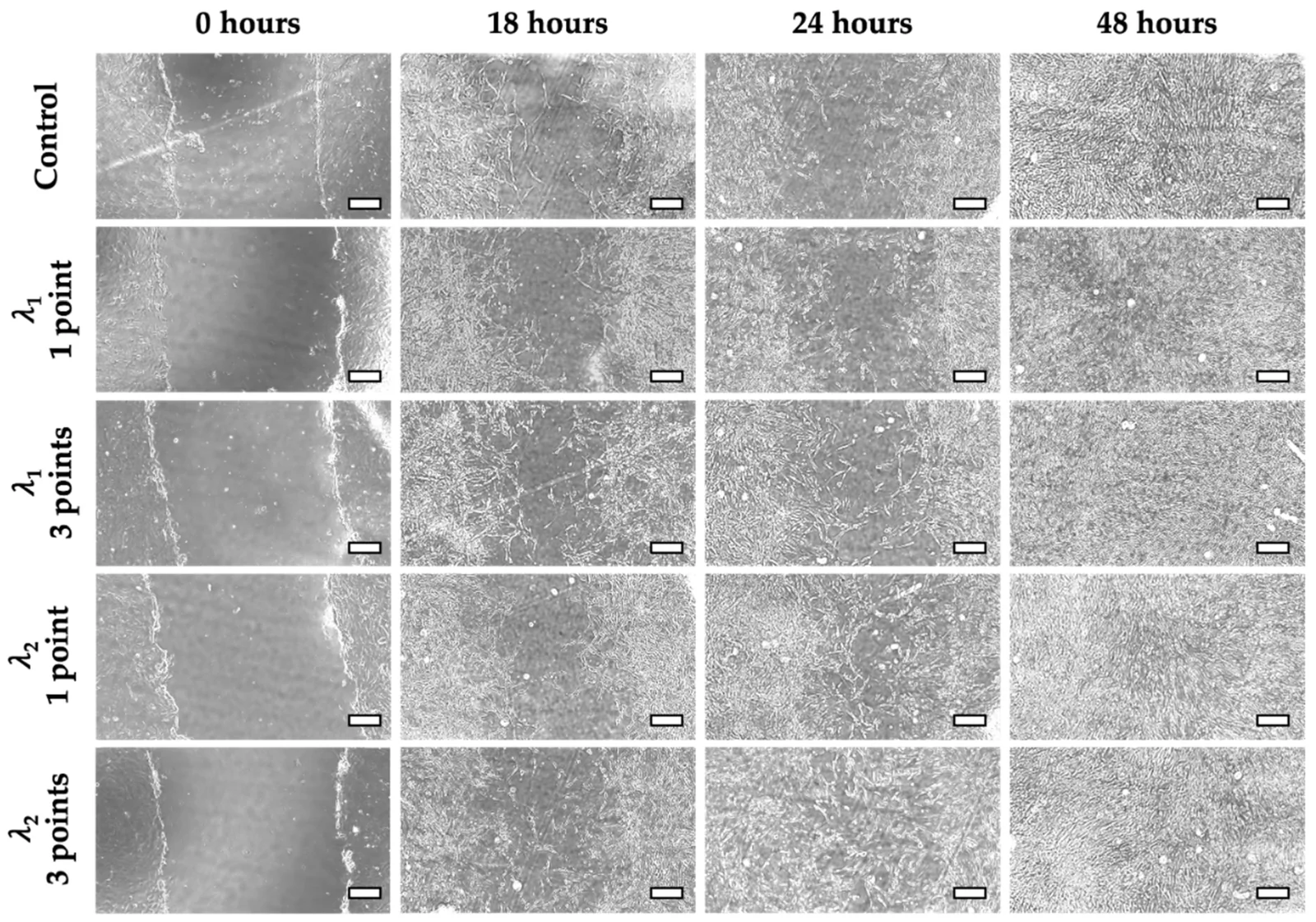
Dynamic observation of a scratch closure among the studied groups in the primary culture of fibroblasts. ${\lambda_{1}=1560\;nm,\;\lambda }_{2}=1450\;nm$. Scale bar: 200 µm.

Scratch closure dynamics were assessed in primary fibroblast monolayers using the scratch assay, a commonly used method for evaluating wound closure behavior in vitro (Figure 3). The method is based on creating a linear defect in a confluent cell monolayer and monitoring the reduction in the cell-free area over time. Because cell proliferation was not pharmacologically inhibited in the present experiment, scratch closure should not be interpreted as a measure of migration alone, but rather as the combined result of cell migration and proliferation.

**Figure 4:**
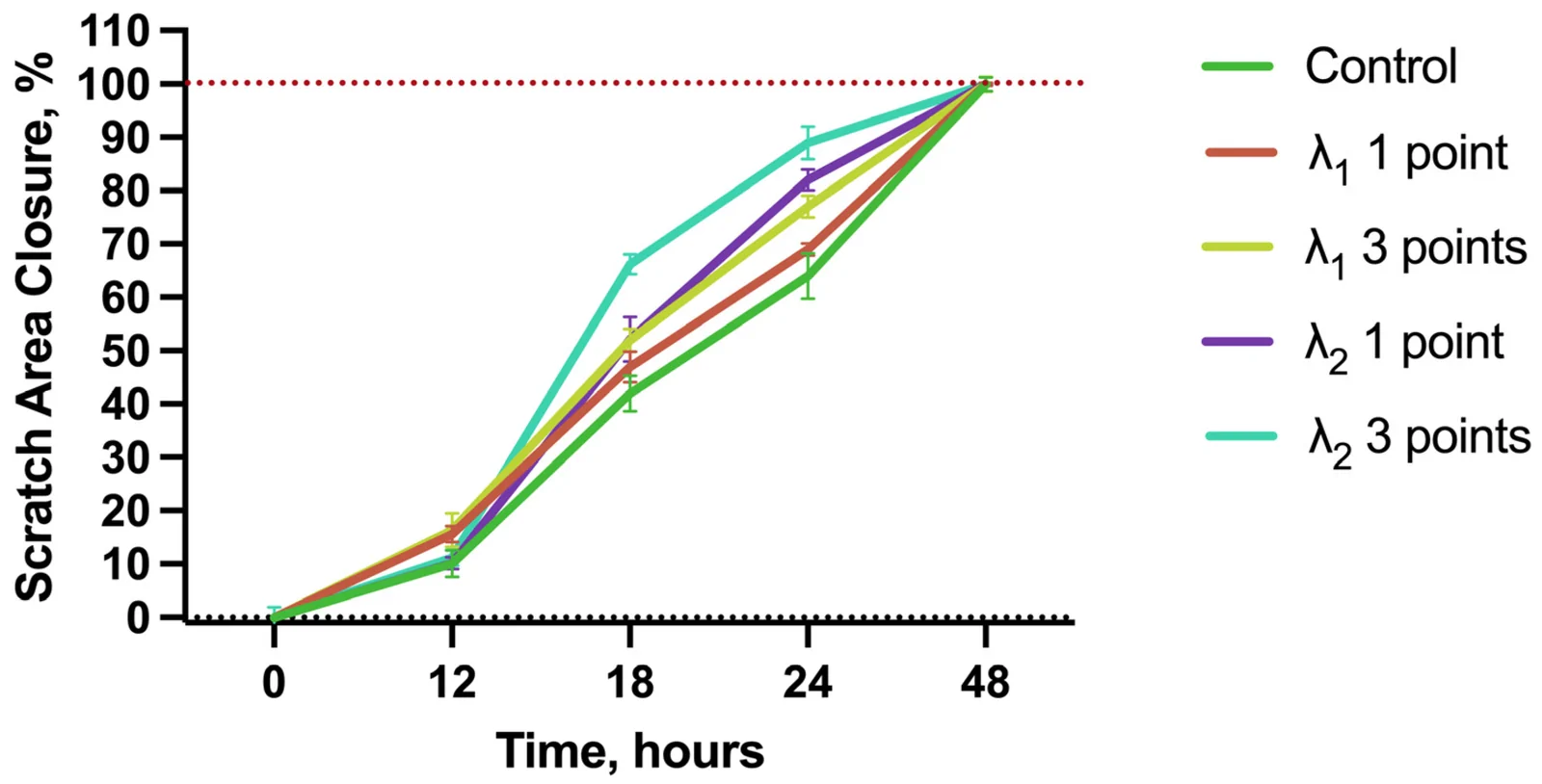
Kinetic curves of a scratch closure among the studied groups in the primary culture of fibroblasts after the laser treatment in 1- and 3-point regimes; ${\lambda_{1}=1560\;nm,\;\lambda }_{2}=1450\;nm$. Data shown as mean of a scratch closure ± SD for 3 independent experiments. Red and black dashed lines indicate the complete and absent scratch closure, respectively.

Kinetic curves of scratch closure in primary fibroblast monolayers among the studied groups are shown in Figure 4.

Already at early time points, especially at 12–18 h, irradiated fibroblast cultures showed accelerated scratch closure compared with the non-irradiated control. The most pronounced effect was observed at 18 h in the group irradiated with the second wavelength using the three-point exposure mode. By 48 h, most irradiated cultures had reached almost complete scratch closure, whereas the control group retained a residual cell-free area. These findings indicate that selected laser irradiation conditions accelerate fibroblast monolayer repair in vitro. However, given the design of the assay, this effect should be interpreted as enhanced scratch closure dynamics rather than as isolated stimulation of cell migration.

### 2.3. Proliferative Activity of the Fibroblasts: Ki-67

The Ki-67 protein is a universal marker of cell proliferation and is widely used in cell biology, oncology, and experimental medicine to assess the intensity of cell division. Ki-67 expression is observed in all active phases of the cell cycle (G1, S, G2, and M), whereas it is completely absent in the resting phase (G0), which makes this protein a reliable marker of cell proliferation.

**Figure 5:**
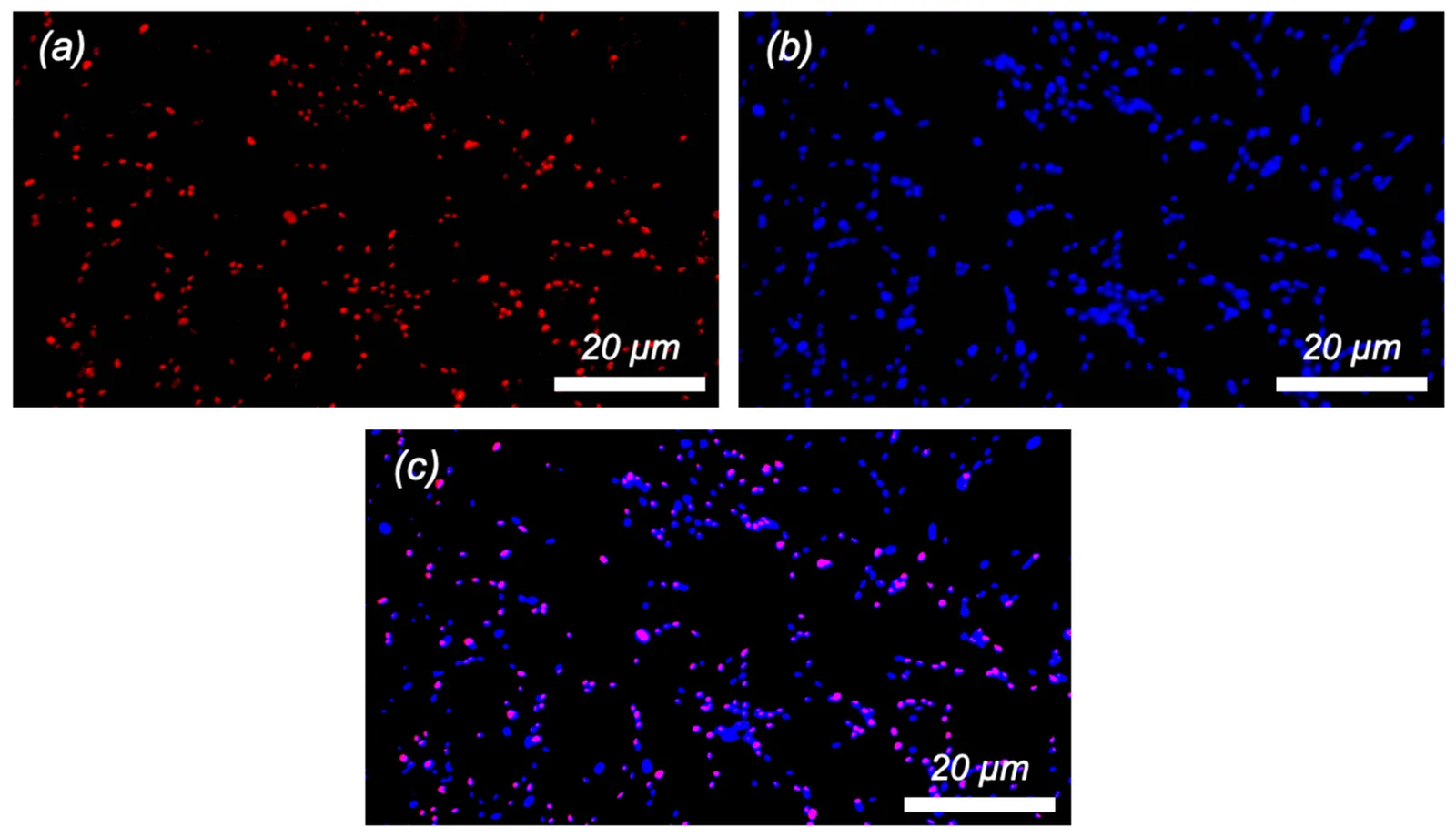
Fluorescent image of (**a**) Ki-67-positive cells (red); (**b**) cells’ nucleus stained with DAPI (blue); and (**c**) combined channels 1 day after the laser treatment: $\lambda_{2}=1450\;nm$, 3 points.

Figure 5 shows examples of fluorescent photographs from which cells were counted and the proliferation index was estimated.

**Figure 6:**
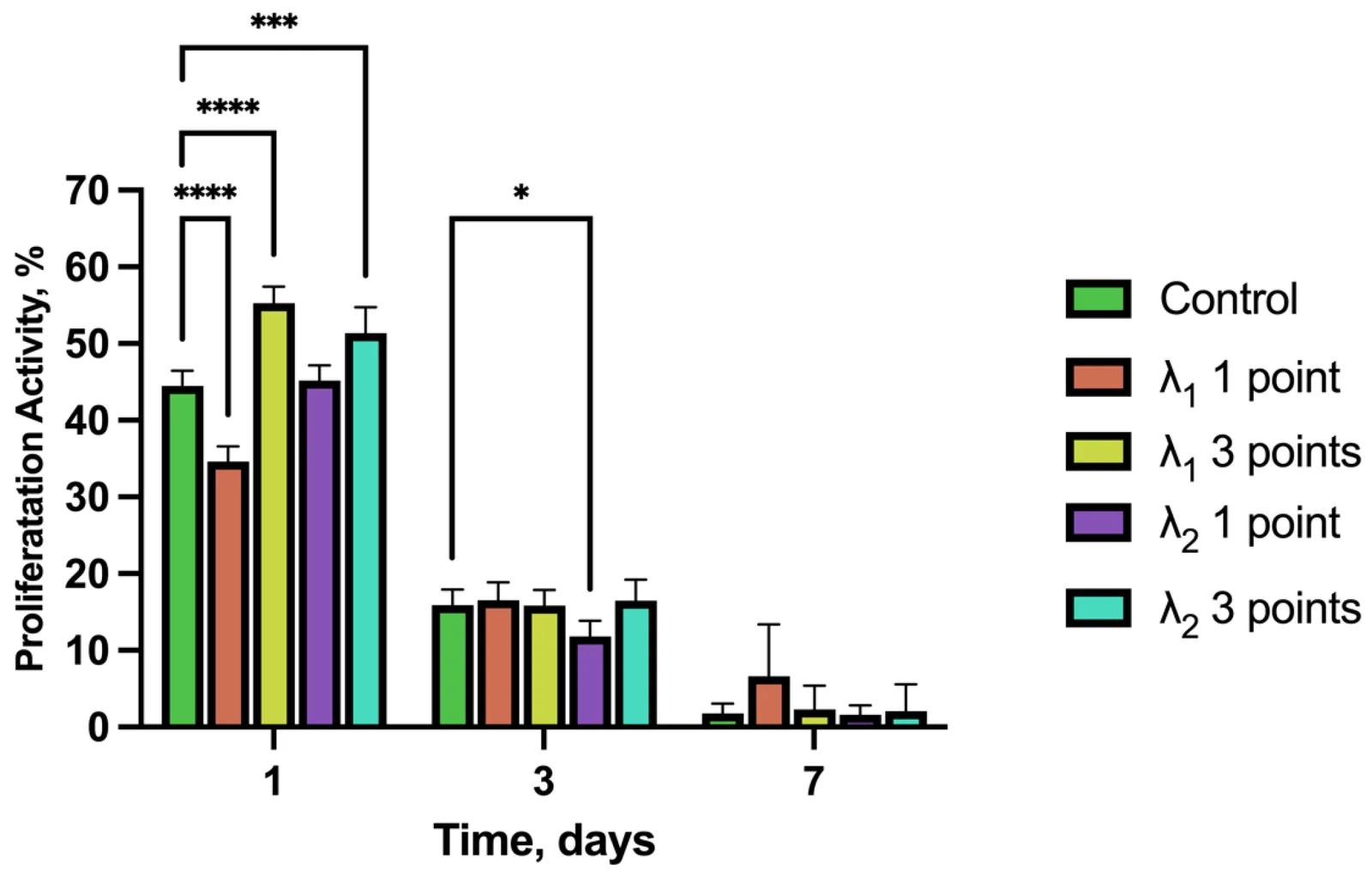
Dynamic changes in the indices of proliferative activity in the cell groups after the laser treatment in 1- and 3-point regimes; ${\lambda_{1}=1560\;nm,\;\lambda }_{2}=1450\;nm$. Data shown as mean ± SD of 3 independent experiments. Statistical significance is indicated as follows: * *p* < 0.05, *** *p* < 0.001, and **** *p* < 0.0001 vs. control (ANOVA and Tukey’s HSD tests).

Figure 6 shows a histogram of changes in the index of proliferative activity of cells, estimated by the level of expression of the Ki-67 marker at different time points of the experiment (1, 3 and 7 days after irradiation). According to the data obtained, there is a gradual decrease in the proportion of cells positive for Ki-67, which indicates a decrease in the intensity of cell division over time. On the first day after irradiation, the proliferation index remains at a level close to the control values, but by the third day there is a downward trend among all groups, which becomes more pronounced on the seventh day. Such a steady decrease in Ki-67 expression may be associated with a slowdown in the rate of mitotic activity as the cell culture reaches high density and some cells enter a dormant state (G_0_ phase).

The dynamics of the Ki-67 decrease correlates well with the results of the MTT test, which reflects the metabolic activity of fibroblasts. The calculation of the Pearson correlation coefficient between the proliferation index and respiratory activity indices gave a value of r = 0.99, which indicates an almost linear relationship between these parameters.

### 2.4. Collagen Type II Concentration

**Figure 7:**
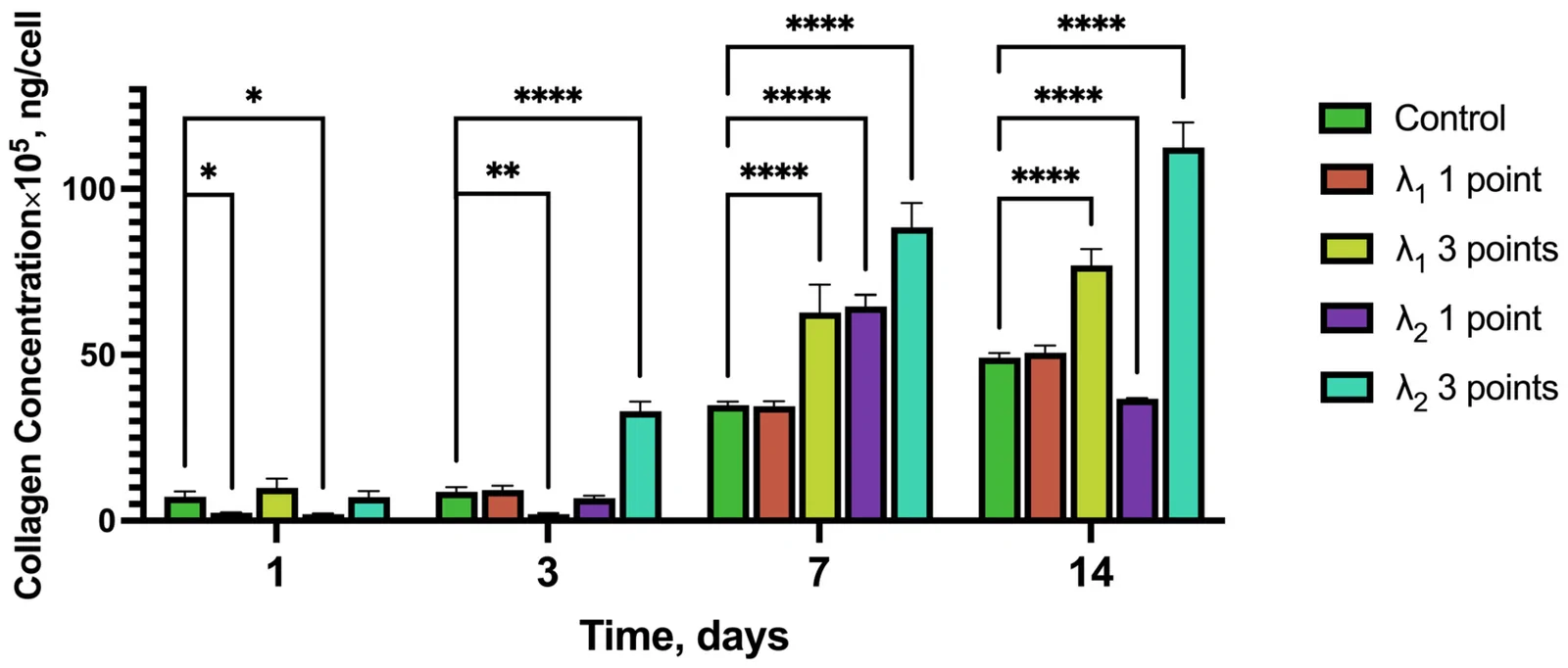
Changes in collagen concentration (normalized per cell) among the fibroblast groups after 1, 3, 7 and 14 days after the laser treatment in 1- and 3-point regimes; ${\lambda_{1}=1560\;nm,\;\lambda }_{2}=1450\;nm$. Data shown as mean ± SD of 5 independent experiments. Statistical significance is indicated as follows: * *p* < 0.05, ** *p* < 0.01, and **** *p* < 0.0001 vs. control (ANOVA and Tukey’s HSD tests).

Figure 7 presents the results of a solid-phase enzyme immunoassay (ELISA) used to quantify the concentration of extracellular matrix (type II collagen).

According to the data obtained, no immediate increase in collagen type II concentration was observed on day 1. By day 7, collagen concentration was significantly increased in the groups irradiated with the second wavelength, regardless of the number of exposure points, compared with the control. By day 14, collagen levels in the second-wavelength single-point group no longer differed significantly from the control, whereas the second-wavelength three-point group maintained the highest collagen concentration and remained significantly different from the control.

Thus, the data do not support a peak of collagen generation on day 3. Instead, they indicate a delayed and irradiation-parameter-dependent increase in extracellular matrix production, with the most persistent effect observed after three-point irradiation with the second wavelength.

### 2.5. In Vivo Studies on the Tympanic Membrane

Based on the in vitro results, the 1450 nm irradiation condition was selected for the in vivo stage because it produced the most consistent effects on scratch closure dynamics and extracellular matrix production.

However, cellular models cannot fully reproduce the complexity of tympanic membrane repair, including epithelial migration, stromal remodeling, scaffold interaction, local inflammation, and multicellular tissue architecture. Therefore, an in vivo pilot experiment was performed to assess whether laser treatment could improve scaffold-assisted repair of chronic tympanic membrane perforations under physiological conditions. The in vivo experiment was conducted in two stages: I) creation of a chronic membrane perforation and II) de-epithelization and laser irradiation. For each animal, one ear (left, *n* = 6) served as a control (control group), while the other one (right, *n* = 6) was exposed to laser radiation (laser group), $\lambda_{2}=1450\;nm$.

**Figure 8:**
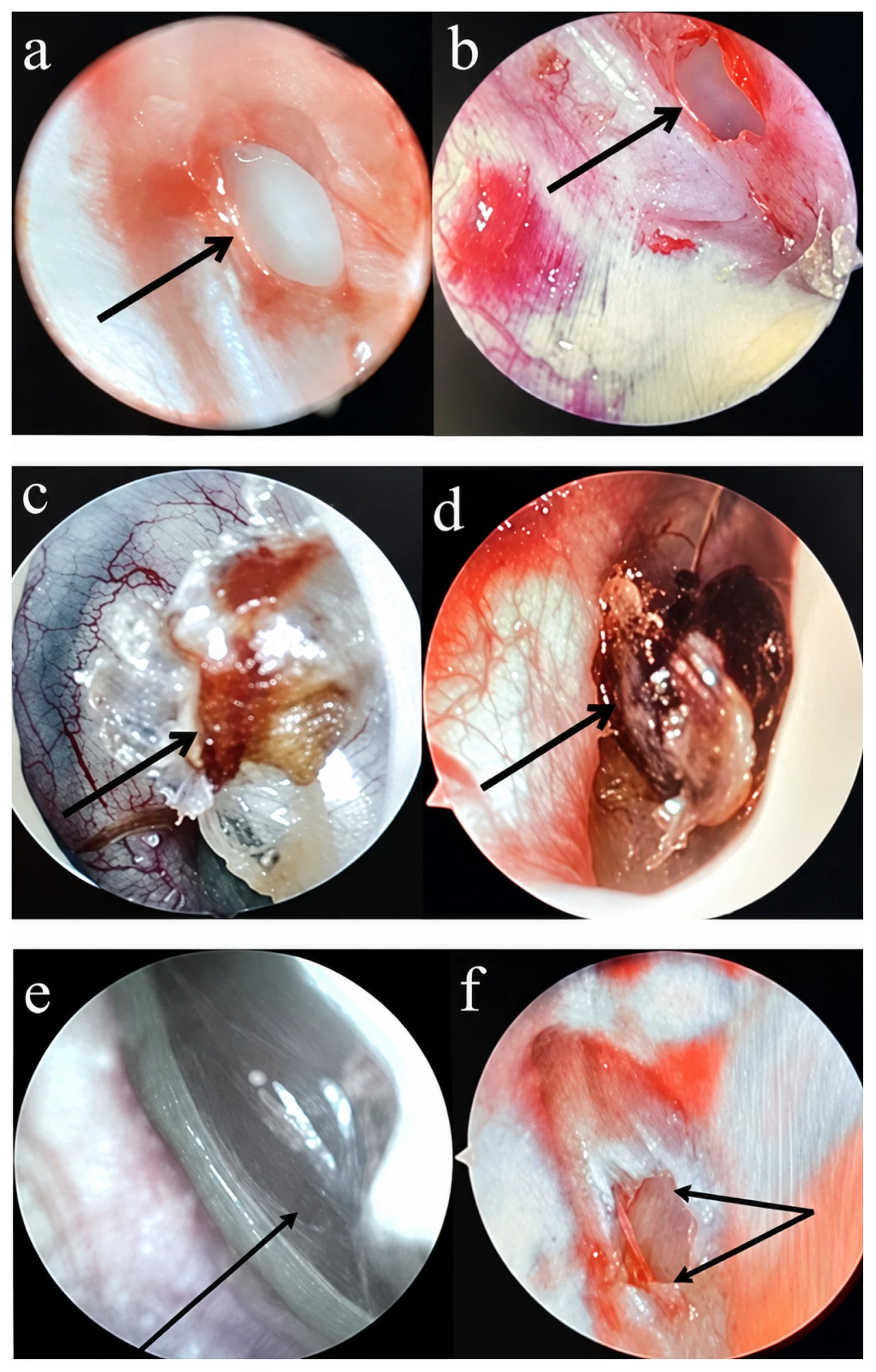
Endo-otoscopic results of the experiment: (**a**) Persistent perforation of the tympanic membrane in laser group before the treatment (indicated by arrow). (**b**) Persistent perforation of the tympanic membrane in control group before the treatment (indicated by arrow). (**c**) The collagen sponge covers the tympanic membrane (indicated by arrow), the perforation is not visible (laser group), 14 days after the treatment. (**d**) The collagen sponge covers the tympanic membrane (indicated by arrow), the perforation is not visible (control group), 14 days after the treatment. (**e**) Restored chinchilla tympanic membrane following laser irradiation in laser group (the scar area is indicated by an arrow), 28 days after the treatment. (**f**) Persistent perforation of the tympanic membrane in the scaffold-only nonirradiated control group untreated control group (the edges of the perforation are indicated by arrows), 28 days.

Following the first stage of the experiment, all animals developed bilateral perforations with thickened margins and no tendency toward size reduction, indicating chronicity (Figure 8a,b). At the 14-day endoscopic examination, preserved collagen sponges were observed covering the perforation surfaces in all animals (Figure 8c,d). The size of the perforation at this time could not be determined without complete removal of the scaffold, which would have disrupted the support for the formation of the keratin bridge.

At 28 days, residual collagen sponges were visualized over the tympanic membrane surfaces in all chinchillas. Complete closure of the tympanic membrane perforation in the right ear at this observation point (day 28) was achieved in all 6 animals. The tympanic membrane appeared gray in color, without signs of inflammation. A thin scar formed at the perforation site, practically indistinguishable from the intact areas. A delicate scar had been formed at the perforation site (Figure 8e).

In the control group (left-sided perforations, *n* = 6), endoscopic examination at all observation time points revealed no cases of closure of the left-sided TM perforations. The perforation margin remained thickened. These data confirm that the perforation created by the described method [29] is persistent (Figure 8f).

The average size of tympanic membrane perforations in this group after collagen sponge removal was 3.32 ± 0.44 mm.

Considering that all perforations were covered with collagen sponge, but closure did not occur, it can be concluded that it does not affect the regeneration of persistent tympanic membrane perforations.

The objectives of our study did not include an assessment of the vibrational function of the tympanic membrane after its healing and auditory function in animals, therefore, such research methods as tympanometry, wideband acoustic immittance, auditory brainstem response testing were not carried out.

## 3. Discussion

The tympanic membrane is a unique three-layered structure that separates the external auditory canal from the middle ear. It plays an important role in sound conduction by transmitting acoustic vibrations to the ossicular chain [30,31]. Conditions such as middle ear infection, extrusion of a ventilation tube, trauma, and other causes can lead to the formation of a tympanic membrane defect. Up to 90% of perforations may heal spontaneously without intervention; however, 10% become chronic. The consequences of persistent perforations include otorrhea and recurrent infections, which can give rise to complications in the middle and inner ear, leading to long-term hearing loss, speech impairment, and, in some cases, the development of cholesteatoma. These and other complications may adversely affect the quality of life, particularly in pediatric patients. Moreover, individuals with TM perforation face numerous limitations in their daily activities due to the elevated risk of infection [7,28,32,33].

The traditional surgical approaches for repairing persistent tympanic membrane defects are myringoplasty and tympanoplasty [34,35]. Considering the variability in individual anatomy and the pathological process within the middle ear, numerous techniques for closing TM perforations have been proposed by various authors, utilizing different autografts, including fascial grafts, perichondrium, cartilage fragments, and multilayered grafts [36,37]. In most cases, surgical treatment is successful; nevertheless, it may have certain drawbacks. The primary disadvantage is incomplete graft integration or graft rejection (reperforation) in the early and late postoperative periods, which in turn necessitates repeat surgical intervention. Furthermore, it should be noted that surgical treatment itself is costly, requires prolonged patient hospitalization, and results in temporary disability [32].

Thus, the search is currently ongoing for a simple outpatient-based procedure for the healing of persistent TM perforations that does not require high-tech surgical equipment and reduces the duration of patient hospitalization.

Biological cells exhibit high sensitivity to changes in their microenvironment, including temperature fluctuations and mechanical stresses. Temporally and spatially modulated laser radiation is capable of generating pulsed-periodic tissue heating, inducing locally non-uniform thermal expansion and the generation of pulsating mechanical stress fields. These controllable factors create specific conditions for cells, enhancing their proliferative activity and stimulating biosynthetic functions. Consequently, laser exposure can be regarded as a tool for the targeted activation of tissue healing and regeneration processes [12]. It is hypothesized that laser radiation may act as a trigger for tissue regeneration in tympanic membrane perforation. For instance, in a study by S. Maleki and colleagues, laser irradiation at wavelengths of 630 nm and 860 nm applied to a post-traumatic tympanic membrane defect resulted in its restoration. A distinctive feature was adequate connective tissue proliferation without excessive fibrosis compared to the control group [38]. The efficacy of low-intensity laser radiation in regenerative medicine is supported by its ability to induce proliferation and differentiation of mesenchymal stromal (fibroblasts, chondrocytes, osteoblasts) and progenitor cells [39].

Previous studies have reported the presence of epithelial stem or progenitor cell populations in specific regions of the tympanic membrane, including the annulus fibrosus, the umbo, and the area along the manubrium of the malleus [25,26,27]. These findings provide a plausible biological basis for the hypothesis that targeted stimulation of these regions may contribute to tympanic membrane repair. In the present study, the laser fiber was directed toward these anatomically relevant zones. However, we did not directly assess progenitor cell activation using marker analysis, histology, or lineage-based methods. Therefore, the involvement of endogenous progenitor cells should be considered a working hypothesis rather than a demonstrated mechanism. To test our hypothesis, we conducted a pilot experimental study to determine the feasibility of closing tympanic membrane defects through laser activation of its tissues.

One of the most prominent findings is the acceleration of scratch closure in fibroblast monolayers after selected laser irradiation conditions. This effect is relevant to tympanic membrane repair, where coordinated cell movement, proliferation, and extracellular matrix remodeling contribute to defect closure. However, because proliferation was not pharmacologically inhibited in the scratch assay, the observed effect should not be interpreted as isolated stimulation of fibroblast migration. Rather, it reflects enhanced wound closure dynamics in vitro, probably resulting from the combined contribution of migration and proliferation. The association between faster scratch closure and increased collagen production further supports the concept of a coordinated repair-related response involving cell redistribution and extracellular matrix remodeling.

The increase in collagen type II concentration was not immediate, but became evident at later time points. The most consistent increase was observed in the second-wavelength groups on day 7, whereas the most persistent effect was detected in the second-wavelength three-point irradiation group on day 14. These findings suggest a delayed and parameter-dependent modulation of extracellular matrix production rather than a short-term peak on day 3. The persistence of elevated collagen levels under multi-point irradiation highlights the potential importance of spatial energy distribution for prolonging the matrix-remodeling response.

In contrast to scratch closure dynamics and matrix production, both metabolic activity (MTT) and proliferation (Ki-67) showed a gradual decline over time. However, this decrease should not be interpreted as an inhibitory effect of laser irradiation. Rather, it reflects the transition of the cell culture to a state of confluence and physiological stabilization, characterized by reduced proliferation and metabolic demand. The strong correlation between metabolic and proliferative indices (r = 0.99) supports this interpretation and indicates that these parameters reflect intrinsic culture dynamics rather than laser-induced suppression. Similar observations have been reported in studies where photobiomodulation promotes early activation followed by normalization of cellular activity.

A key aspect of this study is the demonstrated dependence of the biological response on irradiation parameters. The second wavelength and multi-point exposure consistently produced the most pronounced effects across multiple assays. This finding emphasizes that not only the total delivered energy, but also its spatial distribution and interaction with tissue optical properties determine the outcome of laser stimulation.

The in vivo study was conducted using a 1450 nm laser with an output power of 0.2 Watts. Irradiation of the perforation margins and progenitor zones resulted in the closure of a chronic tympanic membrane perforation in chinchillas. Otovideoendoscopy revealed the formation of precise, thin scars. We hypothesize that reduced inflammation as well as accelerated reparation of TM is attributed to polarization of macrophages into M2 phenotype, associated with anti-inflammatory response [21,24].

The in vitro results demonstrated that irradiation at the second wavelength (1450 nm) using a three-point exposure configuration, which more closely reproduces the spatial irradiation conditions of the intact tympanic membrane, accelerated scratch closure and stimulated collagen production, suggesting enhanced proliferative activity of the treated cells. Consistent with these findings, the corresponding in vivo irradiation protocol was associated with accelerated tympanic membrane regeneration, whereas a persistent perforation remained in the control group even after 28 days of treatment with a collagen sponge. Further studies are required to elucidate the underlying cellular and molecular mechanisms, including the possible involvement of intracellular Ca^2+^ signaling, reactive oxygen species, mitochondrial responses, and downstream proliferative pathways such as MAPK/ERK and Akt, which were not directly assessed in the present study.

The results of this study substantiate the feasibility of further developments in the field of laser-induced regeneration of tympanic membrane perforations. Restoring a tympanic membrane structure that closely approximates the native architecture is also a key prerequisite for the recovery of auditory function.

## 4. Materials and Methods

### 4.1. Primary Fibroblasts Isolation, Cultivation, and Anti-Vimentin Staining

The primary fibroblast culture was isolated from the eardrum of a chinchilla rodent. The animal (one biological donor) was provided by the Central Vivarium of the Sechenov First Moscow State Medical University of the Ministry of Health of the Russian Federation (Sechenov University, Moscow, Russia). Under isoflurane anesthesia, the tissues of the eardrum were endoscopically sampled and immersed in DMEM-F12 nutrient medium (PanEko, Moscow, Russia).

The isolated tissues were thoroughly washed in a sterile phosphate-salt buffer, after which they were subjected to mechanical homogenization to obtain fragments approximately 1 × 1 × 1 mm in size. This approach provided a sufficient surface for cell attachment and subsequent growth, while maintaining cell viability and minimizing damage to the tissue structure.

The obtained fragments were placed in 6-well culture plates and 2 mL of growth medium was poured into each well. The growth medium consisted of a complex nutrient solution including DMEM-F12 (PanEco, Moscow, Russia), 10% fetal bovine serum (Global Kang Biotechnology, Qinhuangdao City, Hebei, China), 1% GlutaMAX (Gibco, Grand Island, NY, USA), 1% of the antibiotic-antimycotic complex Anti-Anti (Gibco, Grand Island, NY, USA), 50 ng/mL of epidermal growth factor (Invitrogen™, Carlsbad, CA, USA), 50 ng/mL basic fibroblast growth factor (Invitrogen™, Carlsbad, CA, USA).

Tissue cultivation continued until stable cell colonies were formed and approximately 60% confluence was achieved, while the growth medium was updated every 2 days to maintain optimal composition and pH. After reaching the required level of consistency, the cells were detached from the surface of the tablet using 0.5% Trypsin-EDTA solution (PanEco, Moscow, Russia), and the remaining tissue fragments were removed.

**Figure 9:**
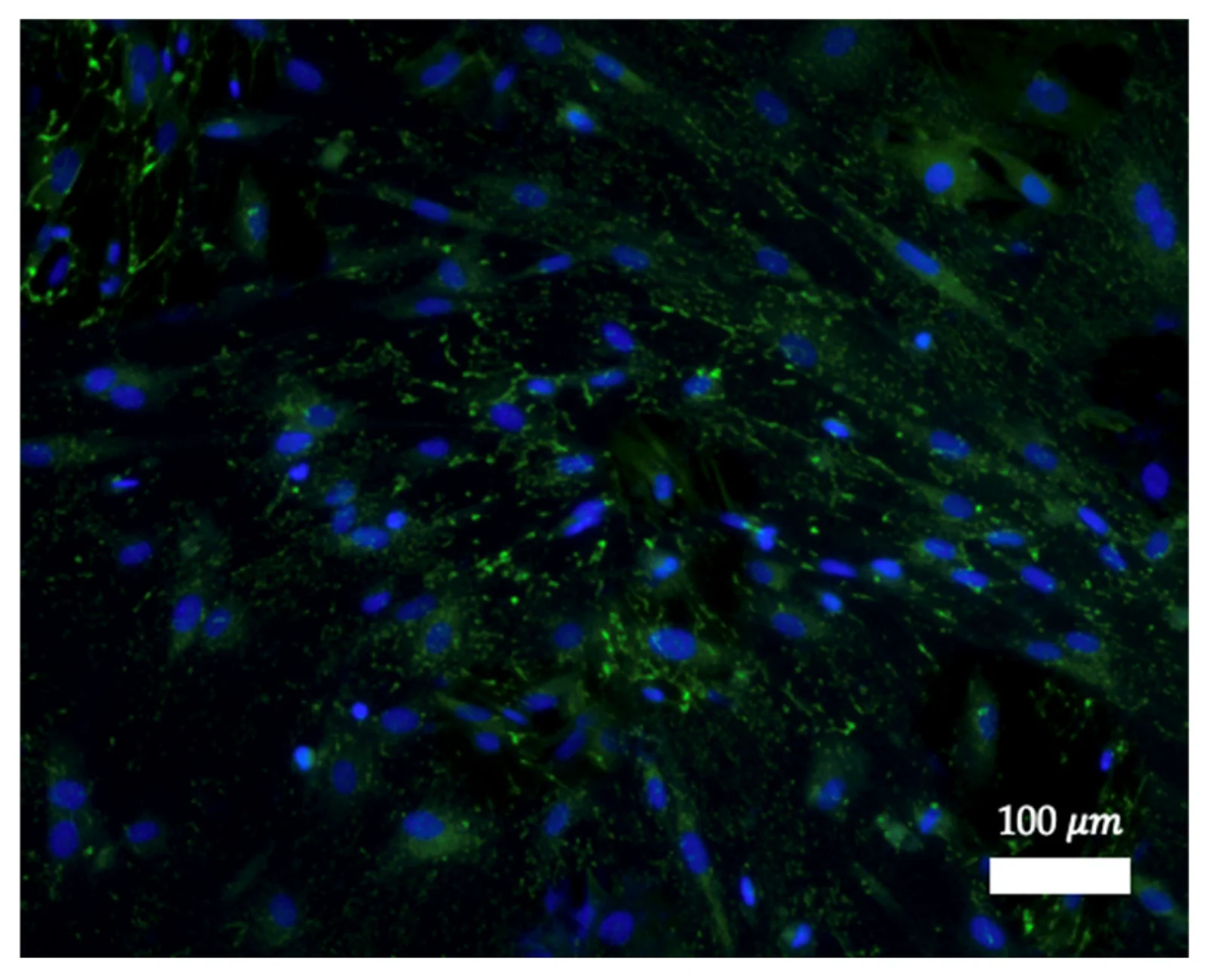
Immunofluorescent staining of vimentin and nucleus of the fibroblast-like cells after the second passage. Green and blue channels are vimentin and DAPI staining, respectively.

To confirm cell identity, they were stained using anti-vimentin antibody conjugated with FITC (Exalpha, Newark, CA, USA). Briefly, cells were cultured in a four-chamber culture plate at a density of 1 × 10^5^ cells/mL. The culture media was removed, and the cells were fixed with 4% paraformaldehyde solution (Solarbio, Beijing, China) with a subsequent gentle washing with Dulbecco phosphate-salt buffer (DPBS, PanEco, Moscow, Russia). Cell membrane permeabilization was performed using 0.5% Triton X-100 solution (Invitrogen™, Carlsbad, CA, USA), followed by washing with DPBS and blocking by addition of 1% bovine serum albumin (BSA; Proliant Biologicals, Ankeny, IA, USA). After an additional washing step, the antibody solution was added at a 1:1000 dilution (antibody:1% BSA) and incubated under dark conditions. Following the final washing step, cells were counterstained with DAPI solution (ServiceBio, Beijing, China) and examined under a fluorescent microscope: $\lambda_{exitation}=490\;nm,\;\lambda_{emmision}=520\;nm.$ As shown in Figure 9, the cells isolated from the tympanic membrane exhibited positive cytoplasmic staining for vimentin.

For subsequent experiments, cells at passage 3 were seeded into 96-well cell culture plates and cultured to the required confluency, reaching approximately 100% confluency for the scratch assay and 50% confluency for the other experiments.

### 4.2. Laser Treatment of the Primary Fibroblasts

As part of the experiment, it was planned to study the effect of laser radiation of two infrared wavelengths: $\lambda_{1}=1560\;nm$ (IRE-POLUS, Moscow, Russia) and $\lambda_{2}=1450\;nm$ (Lantha-Milon, Moscow, Russia). The selected laser radiation modes for both near-IR wavelengths were determined based on preliminary mathematical modeling of laser-tissue interactions at different wavelengths, as well as a pilot study including immediate histological examination of eardrum tissue after laser irradiation [8].

**Figure 10:**
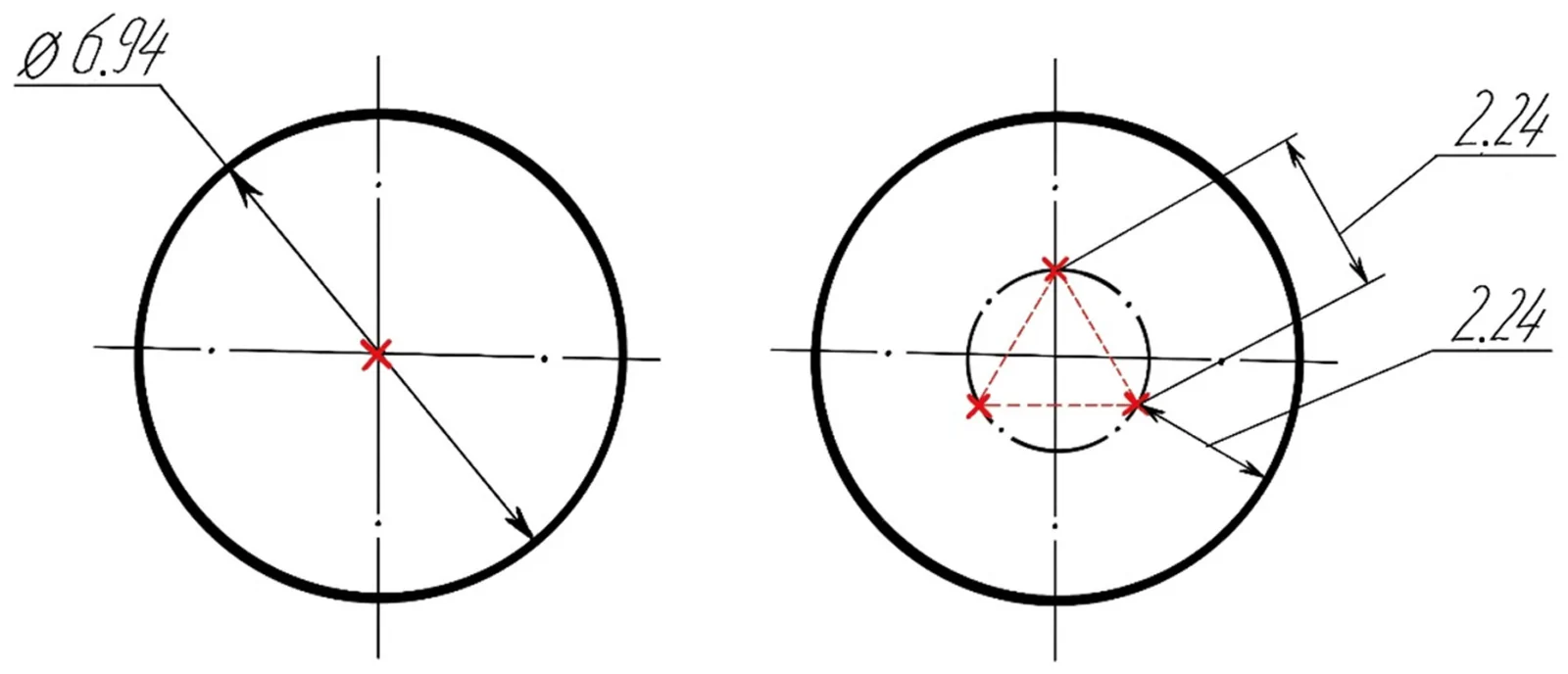
The scheme of irradiation of wells. Red crosses indicate the optical fiber guidance points for one and three irradiation points. Numbers in the scheme have units of millimeters. Red crosses indicate the positions of the laser irradiation points in a well, while arrows indicate the distance between the points and the diameter of the well.

To study the dependence of the studied biological parameters of cells on the number of laser exposures, wells were irradiated in two modes. The distribution schemes of the irradiation zones are shown in Figure 10.

In the case of single-point irradiation, the fiber optic fiber was brought strictly to the center of the well, ensuring a uniform distribution of the light spot over the surface of the cellular monolayer. This arrangement made it possible to concentrate the region of maximum laser-induced thermal stresses within a single well. For the three-point irradiation condition, the spatial distribution of the laser radiation emitted from the optical fiber was calculated based on the experimentally determined scattering profile, and the coordinates of the three irradiation zones were selected to minimize overlap between adjacent exposure areas [12].

Each irradiation point was exposed for 30 s, resulting in a total irradiation time of 90 s for the three-point irradiation scheme. Although the total energy delivered to the experimental area was therefore higher than in the single-point (30 s) condition, the irradiation zones were spatially separated to prevent cumulative overexposure of individual cells; thus, the three-point configuration primarily increased the number of cells exposed rather than increasing the local radiation dose received by cells within the individual irradiation zones. The pumping power of the laser was maintained at 200 mW in all series of experiments, which corresponded to the selected range of therapeutic parameters and did not lead to thermal damage to cells.

### 4.3. MTT Test

To perform the MTT test, the nutrient medium was removed from the wells and washed twice with a solution of Dulbecco phosphate-salt buffer (DPBS, PanEco, Moscow, Russia).

Next, the MTT working solution was prepared by diluting the required amount of 4,5-di methyl thiazol-2-yl)-2,5-diphenyltetrazolium bromide (Neofroxx, Mumbai, India) at a concentration of 5 mg/mL in pure DMEM medium (PanEco, Moscow, Russia).

The MTT working solution was poured into the test wells in a volume of 100 µL and incubated at 37 °C, 5% CO2 for four hours.

After reaching the required incubation time, the MTT solution was removed, and 100 µL of DMSO was added (Servicebio, Beijing, China). Then, for 5 min, the culture plates were left on an orbital shaker at room temperature (RPM = 200) to completely dissolve the crystalline formazane.

The colorimetric analysis was performed using an AMR-100 microplate reader (Allsheng, Hangzhou City, China) at a wavelength of λ = 590 nm.

Metabolic activity was calculated using the following equation:

Metabolic Activity, % = (OD_sample_ − OD_blank_)/(OD_control_ − OD_blank_) × 100%, (1)

where ${OD}_{sample}$—optical density of a test sample; ${OD}_{blank}$—optical density of a blank well; ${OD}_{control}$—absorbance of the control wells (not exposed to laser radiation).

The results are presented as mean ± standard deviation (SD) from three independent experiments, with five technical replicate wells per experimental group in each experiment. Two-way ANOVA was used to assess statistical significance and compare the experimental groups with the control group.

### 4.4. Scratch Test

To conduct the scratch assay, fibroblasts were grown until a dense monolayer was reached (*n* = 5 for each group) onto the bottoms of 96-well culture plates, after which a linear gap of comparable width, approximately 360–370 µm, was created in each well using a sterile pipette tip.

Cell cultures were fixed and changes were documented using a phase contrast microscope. The ImageJ program (v1.54g) with the Wound Healing Size Tool plug-in, specially designed for analyzing scratch test results, was used for a quantitative processing of the obtained images. The data were presented as the mean ± standard deviation of three independent experiments.

### 4.5. Ki-67 Staining and Fluorescent Microscopy

The fixation of the studied cells was carried out as follows: 100 µL of a 4% paraformaldehyde solution (Solarbio, Beijing, China) was added to the corresponding wells (without growth medium and washed with phosphate-salt buffer), kept at room temperature for 15–20 min.

After removing the paraformaldehyde from the wells, the cells were rinsed three times with a 300 µL phosphate-salt Dulbecco buffer for each wash to completely remove the remnants of the fixing solution.

Permeabilization was performed by adding 0.5% Triton X-100 solution (Invitrogen™, Carlsbad, CA, USA), followed by incubation for 15 min at room temperature. After the time had elapsed, the solution was removed by washing once with a phosphate-salt buffer in a volume of 300 µL (10–15 min).

To block the nonspecific binding of antigens, fixed and permeabilized cells were treated with a 1% solution of bovine serum albumin (Proliant Biologicals, Ankeny, IA, USA) in a volume of 100 µL, kept at room temperature for 60 min. After treatment, the wells were washed with a solution of phosphate-salt buffer twice (300 µL, 10–15 min).

A stock solution of mouse monoclonal antibodies Anti-Ki67 antibody [B56] (Abcam, Cambridge, UK) with a concentration of 0.997 mg/mL was diluted in 1% bovine serum albumin solution in a ratio of 1:1000 to obtain a working solution suitable for immunofluorescence staining. Then 50 µL of the working solution were added to wells with cell samples. The samples were incubated with primary antibodies at a temperature of +4 °C for 12 h. At the end of incubation, the wells were washed with a DPBS.

A stock solution of Goat Anti-Mouse IgG H&L (Alexa Fluor^®^ 555) secondary antibodies (Abcam, Cambridge, UK) with a concentration of 2 mg/mL was diluted in 1% bovine serum albumin solution in a ratio of 1:1000 to obtain a working solution. After that, 50 µL of the solution were added to each well. Incubation with secondary antibodies was performed at room temperature for 90 min in light-protected conditions to prevent photodestruction of the fluorophore. At the end of incubation, the wells were washed three times with DPBS in a volume of 300 µL.

All nuclei were stained using a ready-made DAPI solution (ServiceBio, Beijing, China) in accordance with the manufacturer’s instructions. Briefly: 25 µL of the working solution was poured into the test wells, kept in the dark for 8–10 min at room temperature. After that, the solution was removed by washing twice with phosphate-salt buffer.

The cell nuclei (DAPI visualization) were visualized at an excitation wavelength of 340 nm and emission wavelength of 454 nm. The fluorescent images of the secondary antibodies (Ki-67 visualization) were obtained at the excitation wavelength of 555 nm and emission wavelength of 565 nm.

For each condition, at least 6 randomly selected fields of view were captured, evenly distributed across the well surface while avoiding edge regions. Imaging was carried out at a fixed magnification (×20) with separate acquisition of fluorescence channels.

From the data obtained for each well with cells, in accordance with the day of observation and exposure parameters, the index of proliferative activity was calculated using the following formula:

PI, % = N_ki67_/N_DAPI_ × 100%, (2)

where N_ki67_ is the number of cells in the field of view with a positive Ki-67 signal; N_DAPI_ is the total number of cells in the field of view.

Ki-67 immunofluorescence staining was performed in three independent experiments for each experimental group, with 5 technical replicates per group in each experiment. The results are presented as mean values ± standard deviation (SD), with error bars representing the standard deviation. Statistical significance between the experimental groups and the control group was assessed using two-way ANOVA.

### 4.6. Collagen II Concentration Measurements: ELISA

The samples were collected as follows: the wells under study were washed twice with phosphate-salt buffer. Next, 50 µL of 0.5% TWEEN^®^ 20 solution (Sigma-Aldrich, Saint Louis, MO, USA) was poured into each well and left on an orbital shaker at room temperature for 5 min. After that, using a sterile tip for the pipette dispenser, the remains of cells and cell matrix were removed from the bottom and resuspended. The obtained lysates were used to determine the concentration of collagen. As the samples were analyzed at a 1:2 dilution, the measured collagen concentrations were recalculated to account for the dilution factor.

The determination of collagen concentration type II by enzyme immunoassay (ELISA) was carried out in accordance with the protocol of the manufacturer of the ELISA Kit for Collagen Type II (COL2) (Cloud Clone, Madrid, Spain), based on the principle of the “sandwich” method.

**Figure 11:**
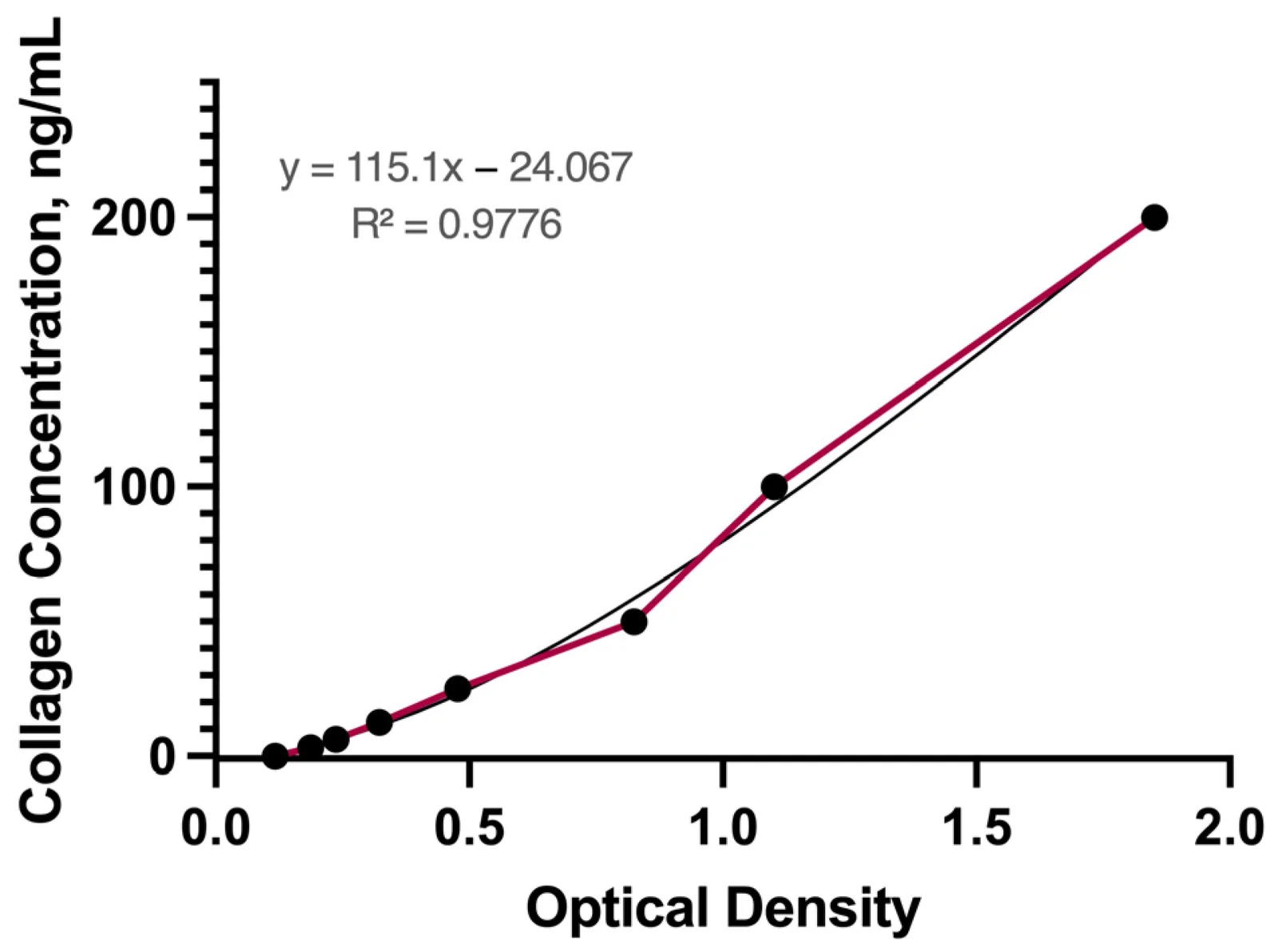
Calibration curve based on measurement results of the standard collagen samples. Red and black lines indicate the experimental and fitted curves, respectively.

The collagen concentration in the studied samples was determined by comparing the optical density values with a calibration curve based on the standard solutions (provided by the manufacturer). The calibration curve is shown in Figure 11.

To account for differences in cell density, the measured collagen concentration was normalized to the corresponding mean cell concentration (the cells were counted using the trypan blue technique) for each group and time point, yielding the collagen production per cell. The results were given as the mean of five independent measurements with a standard deviation.

The data is shown as mean ± SD of three independent experiments, with five technical replicates for each group.

### 4.7. Statistical Analysis

Statistical analysis of the experimental data was carried out using the GraphPad Prism (v 10.3.1 (464)). For each experimental point, calculations were performed based on three independent repeats, which ensured the reliability and reproducibility of the results. All data was preprocessed to determine the mathematical expectation and standard deviation. Statistically significant differences in metabolic activity, proliferative activity, and collagen concentration between the experimental groups and the control group were assessed using two-way analysis of variance (ANOVA), followed by Tukey’s post hoc test for multiple comparisons.

### 4.8. Experimental Study on the Tympanic Membrane

A comprehensive analysis of the available literature indicates that the chinchilla represents the most appropriate animal model for reproducing chronic tympanic membrane perforation. The key advantage over rats and guinea pigs is the wide external auditory canal, which enables simultaneous insertion of an endoscope and surgical instruments. Furthermore, there is a higher similarity in the anatomy of TM and middle ear structures of chinchillas and humans [40,41,42,43].

A total of six male chinchillas, aged 6 months and weighing between 500 and 700 g, were included in the experimental groups. Males were specifically selected to eliminate the potential influence of hormonal fluctuations on the purity of the experimental results. All surgical procedures and animal monitoring were carried out under standard vivarium conditions.

Animal preparation for the experiment included a 12 h fasting period prior to the initiation of procedures. Fifteen minutes prior to general anesthesia induction, premedication was administered via subcutaneous injection of atropine sulfate at a dosage of 0.05 mg/kg. Anesthesia was subsequently induced through intramuscular administration of a combined preparation consisting of Zoletil (10 mg/kg) and xylazine (0.15 mL/kg). Dosages were selected as minimally effective to ensure adequate analgesia during short-term traumatic interventions.

For intraoperative visualization, a 0° rigid endoscope with a diameter of 2.7 mm (Karl Storz, Tuttlingen, Germany) was used. All surgical manipulations were carried out with instruments from a standard otosurgical set, including a microneedle, ear microforceps, and vacuum aspirator tips ranging from 1.4 to 2.0 mm in diameter.

The experiment was conducted in two stages.

During the first stage, persistent bilateral tympanic membrane perforations (diameter 5.25 ± 0.22 mm) were created in all animals using a methodology previously developed by the Department of Otorhinolaryngology at Sechenov University [29]. A 3–4 mm diameter perforation is created in the posteroinferior quadrant using a microneedle. Four radial incisions are then made around the perforation using the same instrument at 12, 3, 6, and 9 o’clock, and the resulting triangular flaps are folded inward toward the medial surface of the tympanic membrane. A tympanostomy tube is then inserted into the defect for 4 weeks. The shunt is then removed. The persistent perforation is monitored for another 4 weeks, with any signs of improvement assessed. The perforation is considered persistent if it successfully passes this control period.

**Figure 12:**
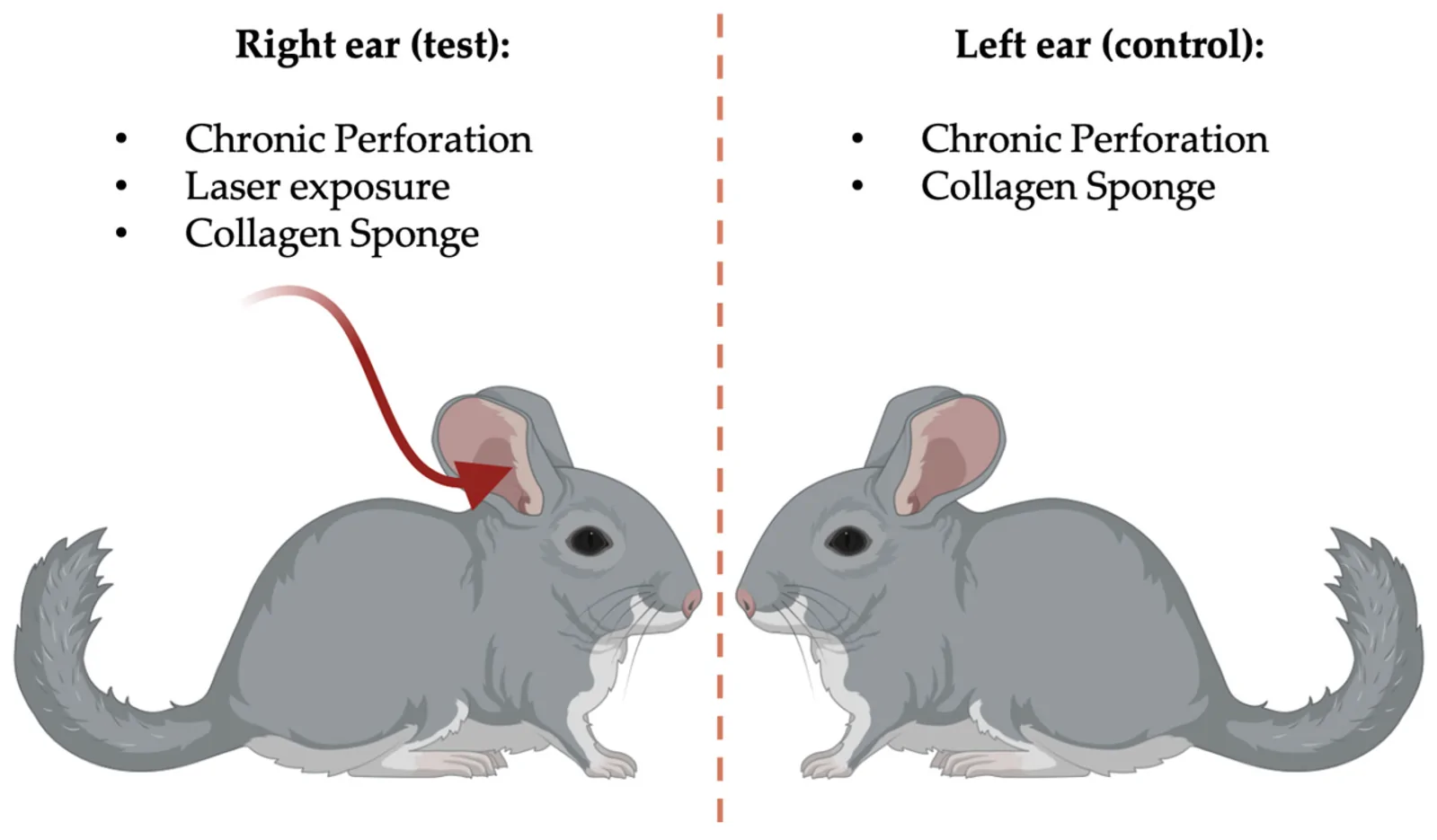
Experimental setup of the in vivo experiment.

In the second stage, all chronic perforations were de-epithelialized and subsequently divided into two groups: a control group (*n* = 6, left ear) and a laser group (*n* = 6, right ear). The experimental setup is represented in Figure 12. The perforations on the right side were irradiated with a 1450 nm diode laser (pulse duration: 100 ms, repetition rate: 1 Hz, power: 0.2 W).

**Figure 13:**
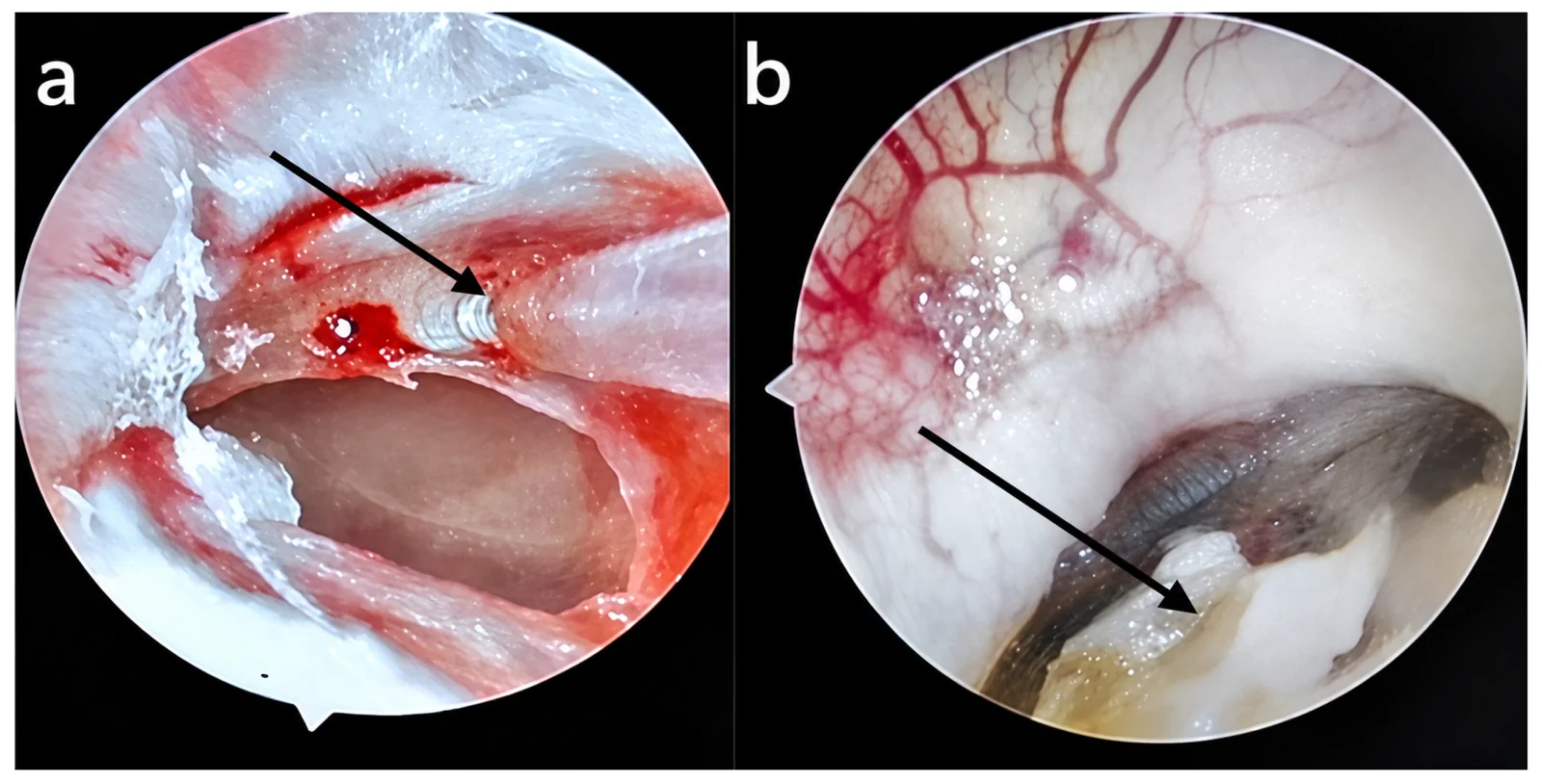
Experimental stages: (**a**) Laser irradiation of the tympanic membrane tissue (one of the regenerative potential zones—annulus fibrosus)—the laser optical fiber is indicated by the arrow. (**b**) Collagen scaffold placed over the perforation of the chinchilla’s tympanic membrane from laser group (indicated by the black arrow).

Using a 0° rigid endoscope for visualization, the tympanic membrane and its perforation with previously freshened margins were identified. The laser optical fiber was then introduced into the external auditory canal and positioned close to, but without contacting, the tympanic membrane surface. The fiber was sequentially directed to the following sites: the malleus zone, the annulus fibrosus zone, and 2–3 points along the perforation margin (Figure 13a). According to the literature, these regions contain progenitor cell populations that may contribute to the regenerative capacity of the tympanic membrane [25,26]. In the present study, irradiation of these anatomically relevant zones was performed based on the hypothesis that targeted stimulation may support repair; however, progenitor cell activation was not directly assessed.

All left-sided persistent tympanic membrane perforations (*n* = 6, left ear) constituted the chronic perforation control group.

According to the literature, tympanic membrane healing begins with the formation of a keratinous bridge across the defect. It serves as a scaffold for the epithelial layer. The regeneration of connective tissue fibers commences only after the bridge is restored [44]. To ease cell migration, various allogeneic scaffolds are employed, which are placed over the tympanic membrane defect to enhance healing [45]. Collagen is a natural polymeric material due to its availability and high biocompatibility [46]. Literature data indicate the absence of rejection reactions with collagenous materials, rendering them optimal scaffolds for tympanic membrane defect closure [47]. Collagen possesses unique biological properties compared to other biomaterials that enable wound healing. Specifically, collagen sponges have demonstrated efficacy in absorbing tissue exudate upon wound contact, ensuring a moist environment and preventing infectious processes and mechanical damage [48].

Consequently, all tympanic membranes (both control and laser groups) were covered with a collagen sponge utilized as a scaffold. Its circumference was 1–2 mm larger than the perforation (5.25 ± 0.22 mm) (Figure 13b).

During the postoperative follow-up no pharmacological therapy was administered.

Endoscopic monitoring of regeneration was performed in all animals. On day 14, the presence or absence of signs of inflammation of the tympanic membrane (hyperemia, edema, vascular injection) was recorded.

On day 28, perforation closure and the severity of cicatricial changes in the tympanic membrane were assessed.

## 5. Conclusions

This pilot study suggests that low-intensity infrared laser irradiation can modulate cellular processes relevant to tympanic membrane repair and may enhance collagen sponge-assisted closure of chronic tympanic membrane perforations in a chinchilla model. The combination of in vitro assays and an in vivo chronic perforation model allowed us to assess both cellular responses to irradiation and the potential tissue-level relevance of these effects.

In vitro, selected irradiation conditions accelerated scratch closure dynamics in primary chinchilla fibroblast monolayers and increased extracellular matrix production at later time points. The most consistent effects were observed under the 1450 nm irradiation condition and, in particular, with multi-point exposure. These findings suggest that the biological response depends not only on wavelength, but also on the spatial distribution of irradiation. At the same time, metabolic activity and Ki-67 expression decreased over time, most likely reflecting culture maturation, increasing confluence, and contact inhibition rather than cytotoxicity or a generalized inhibitory effect of laser exposure.

In vivo, complete closure of chronic tympanic membrane perforations was observed in all laser-treated ears, whereas scaffold-only control perforations remained unclosed during the observation period. Since collagen sponge scaffolds were used in both groups, the regenerative outcome should be interpreted as an effect of laser treatment in the context of scaffold-assisted repair rather than as a result of laser irradiation alone. This distinction is important for the translational interpretation of the findings and for the design of future preclinical and clinical studies.

Overall, the results support further investigation of laser-assisted, scaffold-supported tympanic membrane repair as a minimally invasive strategy for chronic perforations. However, the present study should be regarded as a pilot investigation. The small number of animals, the limited duration of follow-up, and the absence of direct histological or molecular confirmation of the proposed mechanism restrict the strength of the conclusions. In particular, the possible involvement of endogenous tympanic membrane progenitor cells remains a plausible hypothesis based on the previous literature, but was not directly demonstrated in the present work.

Additional work is also required to optimize irradiation parameters and to determine whether the observed anatomical closure is accompanied by restoration of tympanic membrane architecture and auditory function. These steps will be necessary before the proposed approach can be considered for clinical translation.

## Data Availability

The raw data supporting the conclusions of this article will be made available by the authors on request.
